# Neuroanatomy and transgenic technologies

**DOI:** 10.3389/fnana.2014.00157

**Published:** 2015-01-21

**Authors:** Alexander C. Jackson, Chen Liu, Makoto Fukuda, Michael Lazarus, Laurent Gautron

**Affiliations:** ^1^Department of Physiology and Neurobiology, University of ConnecticutStorrs, CT, USA; ^2^Division of Hypothalamic Research, Department of Internal Medicine, University of Texas Southwestern Medical CenterDallas, TX, USA; ^3^Baylor College of Medicine, Childrens Nutrition Research CtHouston, TX, USA; ^4^International Institute for Integrative Sleep Medicine (WPI-IIIS), University of TsukubaTsukuba, Japan

**Keywords:** mouse models, transgenic, tracing, morphology, neurochemistry

Gerald Edelman once wrote: “If someone held a gun to my head and threatened oblivion if I did not identify the single word most significant for understanding the brain, I would say ‘neuroanatomy.’ Indeed, perhaps the most important general observation that can be made about the brain is that its anatomy is the most important thing about it” (Edelman and Tononi, 2000).

Neuroscientists increasingly rely on techniques enabling them to manipulate genes in defined cell populations in the brain. In particular, engineered transgenes, which encode a variety of fluorescent reporter proteins, can be inserted into the genome or delivered into desired brain regions using viral vectors, thereby allowing the labeling of molecularly-defined populations of neurons or glial cells (Callaway, 2005). Transgenic technology can also be used to selectively delete genes in genetically-targeted cell populations (Nagy, 2000) or bi-directionally modulate electrical excitability using optogenetic or chemogenetic techniques (Aston-Jones and Deisseroth, 2013). One of the primary advantages of using transgenic reagents is to simplify the identification of targeted populations of neurons and their projections, which can be laborious using traditional techniques in neuroanatomy. In this research topic, we will be focusing on the application of transgenic technology to neuroanatomical questions and have collected up-to-date reviews and original articles that demonstrate the versatility and power of transgenic tools in advancing our knowledge of the nervous system. Kou et al. (2013) used a GAD67-GFP transgenic mouse to examine changes in components of GABAergic neurotransmission among neuronal populations in the dorsal cochlear nucleus, in a mouse model of noise-induced hearing loss. This was complemented by the article of Zhang et al. (2013), characterizing the efferent projections of adenosine A_2A_ receptor-expressing neurons in the nucleus accumbens using a virally-mediated anterograde tracing method in adenosine A_2A_ receptor-cre mice. In addition to its utility in tagging and tracing targeted neuronal populations, transgenic technology can be applied to the study of morphological and neurochemical changes occurring in the brains of animals lacking a specific gene. For instance, Xu et al. (2014) examined changes in CART expression in neurons of the Edinger–Westphal nucleus in leptin receptor deficient mice.

Needless to say, the study of the peripheral nervous system has also greatly benefited from the aforementioned transgenic tools (Braz et al., 2014). Several articles included in this research topic focused on the peripheral nervous system. We highly recommend the reading of the article by Le Pichon and Chesler (2014), as it is a comprehensive and thoughtful review entirely dedicated to mouse models useful for the manipulation and categorization of somatosensory neurons in the dorsal root ganglion. Many investigators and clinicians are also interested in identifying new ways of delivering transgenes, particularly ones with therapeutic value, to the brain by targeting neurons in the peripheral nervous system. In fact, it has been suggested that virally-mediated gene delivery to the human brain may have the potential to treat numerous neurological diseases (Janson et al., 2002). Three articles in this research topic highlighted the versatility of virally-mediated gene delivery to peripheral pathways. The original articles by Schuster et al. (2014a,b) and Salegio et al. (2014) described the central distribution of intrathecally-delivered adeno-associated viruses expressing reporter proteins. Finally, the article by Jara et al. (2014) focused on approaches for the retrograde labeling of spinal motor neurons using reporter proteins.

In spite of the growing number of sophisticated tools available to neuroscientists, currently available tools greatly limit our ability to collect high resolution images encompassing large areas of the mammalian nervous system (Lichtman and Denk, 2011). The opinion article by Pabba (2013) briefly discussed how the anatomical organization of the central nervous system, with a special emphasis on the amygdaloid complex, has increased in complexity during the course of evolution, while conserving a common basic plan, recognizable across animal species. Henceforth, studying the nervous system of lower organisms is immediately relevant to the understanding of the basic principles governing the anatomical organization of the mammalian nervous system (Amat et al., 2014). This research topic included two original articles focusing on the zebrafish nervous system. Using a zebrafish transgenic line expressing eGFP, Djenoune et al. (2014) described a poorly characterized group of specialized neurons that make contact with the cerebrospinal fluid. This was complemented by an article by Lopez-Schier et al. (Pinto-Teixeira et al., 2013) describing a novel method for the visualization of sensory hair-cell regeneration in the lateral line of transgenic zebrafish larvae using selective plane illumination microscopy (SPIM). The technique described in this latter article offers a unique model for the study of neuroplasticity in a living organism.

In summary, the anatomical complexity of the nervous system remains a subject of tremendous fascination among neuroscientists. Unraveling the myriad cells and circuits in the nervous system is as much a pressing question now as it was over 100 years ago in the time of Cajal and Golgi. In order to tackle this extraordinary complexity, powerful transgenic technologies are continually being developed, improved upon and used in the study of such diverse questions as cell lineage mapping, neural tract tracing, protein trafficking, neuronal excitability and morphological plasticity of dendritic spines and axonal arbors. In addition to giving neuroscientists an update on these rapidly evolving techniques used in neuroanatomy, we hope that the articles included in this research topic will spark new ideas among investigators interested in the “most important thing” about the brain—its anatomy.

## Conflict of interest statement

The authors declare that the research was conducted in the absence of any commercial or financial relationships that could be construed as a potential conflict of interest.
